# Targeting HCV polymerase: a structural and dynamic perspective into the mechanism of selective covalent inhibition

**DOI:** 10.1039/c8ra07346e

**Published:** 2018-12-18

**Authors:** Letitia Shunmugam, Mahmoud E. S. Soliman

**Affiliations:** Molecular Bio-Computation and Drug Design Laboratory, School of Health Sciences, University of KwaZulu-Natal Westville Campus Durban 4001 South Africa; School of Health Sciences, University of KwaZulu-Natal Westville Campus Durban 4001 South Africa soliman@ukzn.ac.za +27 (0) 31 260 7872 +27 (0) 31 260 8048

## Abstract

*Background*: Concerns have been raised over the emerging pandemic status of hepatitis C virus (HCV). Current available drugs lack specificity, stability and potency. The HCV NS5B RNA-dependent RNA polymerase (RdRp) is a vital component in viral replication and is often targeted in antiviral therapies. Recent experimental procedures have led to the discovery of a novel covalent RdRp inhibitor, compound 47, which selectively targets cysteine 366 of the HCV RdRp and exhibits promising pharmacokinetic outcomes. Selective covalent inhibition of HCV is, however, a highly neglected subject in the literature, that is reinforced by the lack of efficient structure-based drug design protocols. In this paper, an atomistic insight into a novel selective approach to inhibit HCV RdRp is provided. *Methodology*/*Results*: Covalent molecular dynamic analyses revealed the inhibitory mechanism of compound 47 on the RdRp. Inhibitor binding induced distinctive internal movements resulting in the disruption of normal physiological interdomain interactions. *Conclusion*: Compound 47 stimulates reorganization of key protein elements required for RNA transcription, thus hampering viral replication as well as disrupting the overall conformation of HCV. This study will open new lines of approach for the design of novel selective inhibitors against HCV as well as other viral families.

## Abstract 2

*In silico* study investigating the interaction between HCV polymerase and selective covalent inhibitor, compound 47.

## Graphical abstract

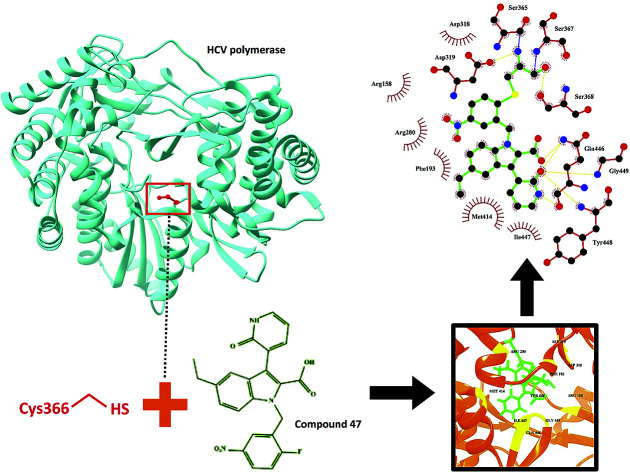

## 1. Introduction

The hepatitis C virus (HCV) is a membrane-bound, hepatotropic RNA virus.^1^ Over the years, following its initial discovery in 1989,^2^ HCV has gained global concern over its ascent to pandemic status. It is thought that at least 3% of the world's population has been infected with HCV, of which a further 30% will go on to develop severe hepatic-related diseases including cirrhosis and hepatocellular cancers.^3^

**Fig. 1:**
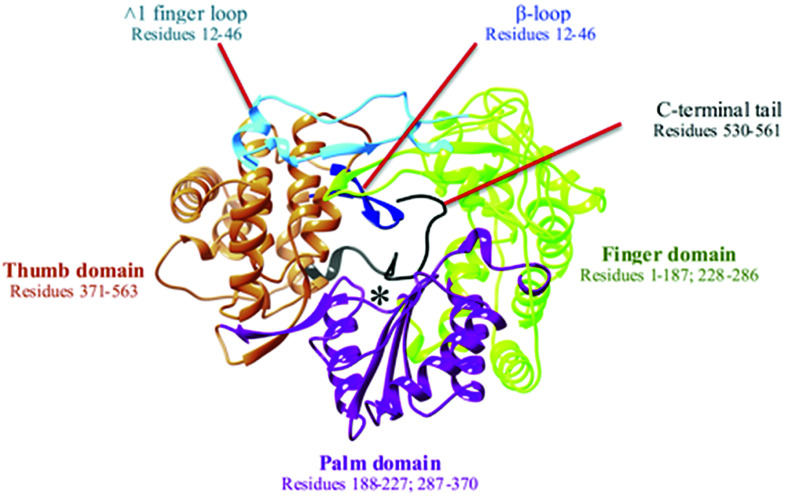
Graphical representation of right-hand X-ray crystallography structure of NS5B RNA dependent RNA polymerase (PDB ID: 3H5S). The active site is denoted by *.

The HCV genome is translated into a polymer consisting of 3010 amino acid residues, which are cleaved by both viral and cellular proteases to generate the proteins necessary for replication and viral assembly.^4,5^ Amid these proteins is the non-structural 5B RNA-dependent RNA polymerase (NS5B RdRp). The RdRp of HCV is a 66 kDa protein comprised of approximately 591 amino acids and is located at the C-terminal of the viral genome. The RdRp occupies a distinguishable three-dimensional structure that adopts a right-handed topology with distinctive domains recognized universally as the finger, palm, and thumb regions^6^ (Fig. 1).

Identified as an essential component of the HCV viral life cycle, RdRp adopts a vital role in the RNA genome replication and transcription. The RdRp has two modes of enzyme activity that correspond to the different conformations. Extensive interdomain contact between the finger and thumb domains facilitate the enzyme's closed “active” conformation, which permits the *de novo* synthesis of RNA for initiation of viral replication. However, the enzyme adopts a more open conformation by displacement of the C-terminal tail which also serves as the linker region and the β-loop. In current research, the open conformation of RdRp is still an ongoing investigation and very little is understood about it at the moment, however, evidence seems to suggest that this state is an indication of enzyme inactivity as intramolecular domain interactions vital for the initiation of replication are disturbed.^7^ Thus, RdRp and its conformation has immense importance in viral replication, and for this reason, it is frequently targeted by the medicinal chemistry researchers and the pharmaceutical industry for the design and development of potent antiviral therapies.^8^

Over the last 20 years, several candidate drug have been discovered and implemented in HCV therapeutics. The drugs are classified into two distinct classes: nucleoside/nucleotide inhibitors (NIs) and non-nucleoside inhibitors (NNIs), categorization is dependent on the mechanism of action.^9^ The NIs serve as imitator substrates for the RdRp to prevent RNA chain elongation through nucleoside triphosphate displacement.^10^ Notable NIs include valopicitabine (Indenix), balapiravir (Roche), mericitabine (Pharmasset/Roche)^11,12^ and uprifosbuvir (Merck & Co).^13,14^ In contrast, NNIs preferably bind to allosteric sites in the RdRp palm or thumb domains, initiating conformational modifications of the enzyme, which hinders its function in the initiation of RNA synthesis.^10^ Examples of NNIs include ABT-072, setrobuvir and dasabuvir.^15,16^

Although the currently available HCV RdRp inhibitors exhibit potent activity against the viral enzyme, their poor chemical stability and relatively high molecular weights have led to challenges arising in their pharmacokinetics especially concerning drug absorption, distribution, biotransformation and elimination.^17,18^ Furthermore, the evolution and adaption of HCV in favour of survival has primed the onset of mutations at numerous sites leading to genetic variability amongst genotypes. A process that is further escalated by RdRp's lack of adequate proofreading capabilities, means that the enzyme is unable to correct mechanistic errors, and this may potentially lead to multiple genetic variations of the virus within a single infected individual.^19^ The generation of HCV mutant forms has actively caused the resistance of the virus to non-selective enzyme inhibitors.^20,21^ In due course, researchers developed a new therapeutic approach that specifically targeted the RdRp of multiple if not all HCV geno- and sub-types, later designated as selective covalent inhibition. In theory, the approach appeared to fulfill all the aspects lacking in current therapies, however, its practicality was certainly questioned because of difficulties encountered during its implementation in drug discovery as a result of scarce availability of competent protocols.^22^

**Fig. 2:**
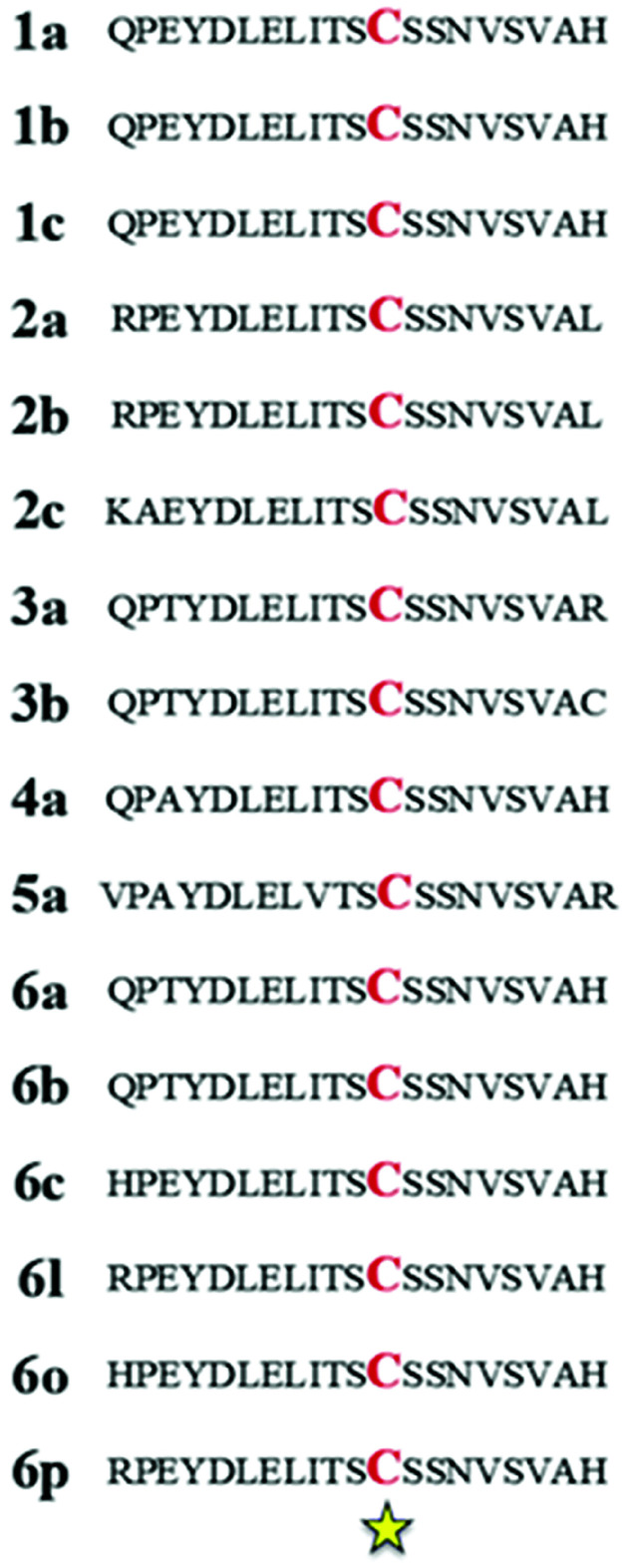
Sequence alignment of NS5B polymerase. Cysteine 366 is conserved within all the genotypes of HCV. C: cysteine (red); yellow star indicates identical residues. The genome sequences for each of the HCV genotypes were obtained using UniProt and subsequently aligned using ClustalW2.^27,28^

Selective covalent inhibition involves the binding of an inhibitor possessing a highly selective nature, to a residue belonging to a serine or cysteine amino acid in the protein of interest. The designated amino acid can be extrapolated from reports in the literature whereby experimental investigations reveal its conservative nature across multiple genotypes of a virus. The resultant bond formed between the two components are thought of as an irreversible drug–protein interaction.^23^ In 2006, Lee *et al.* were the first to identify the importance of cysteine 366 (Cys366) in specific targeting of HCV RdRp.^24^ The study discovered that mutation of cysteine to glycine (C366G) results in drastic loss of wild-type RdRp enzyme activity. These findings disclosed the importance of Cys366 in selective inhibitor binding and full enzymatic functioning of HCV RdRp. The Cys366 amino acid residue is protected across all HCV RdRp sequences known to date (Fig. 2). Thus, Cys366 is an ideal target for obtaining selectivity in covalent HCV inhibition.^24^ However selective covalent inhibition in association with HCV RdRp has not yet been adequately investigated, which is evident by the lack of reports in the literature. This lack of research has negatively impacted the design and discovery of potentially effective HCV antiviral therapies.^25,26^

**Fig. 3:**
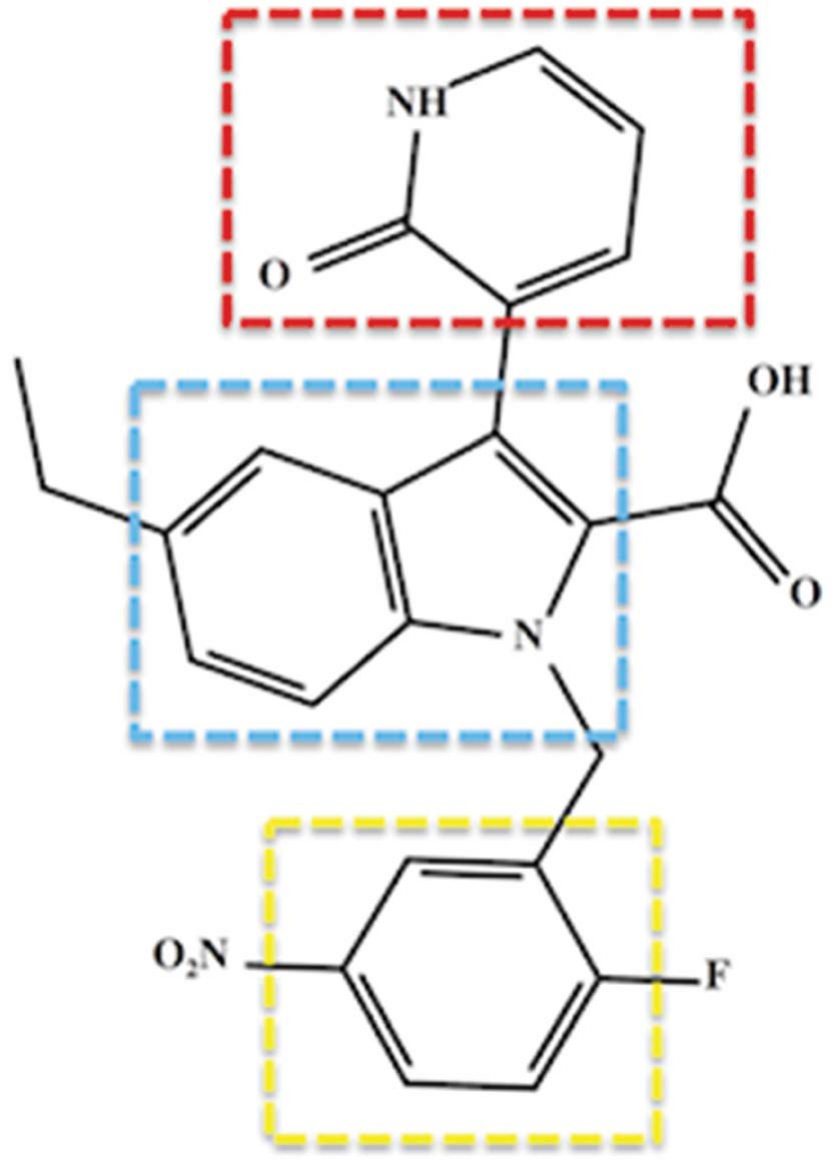
Two-dimensional structural representation of the HCV RdRp covalent inhibitor, compound 47. Red: C-3 pyridone ring; blue: indole core and yellow: benzene ring. The compound exhibited covalent binding to RdRp whereby the thiol group of Cys366 attacked the benzene ring at the *para* position to the nitro group and the fluoro group is released presumably through an aromatic nucleophilic substitution reaction.^6^

Soon after its discovery, Chen *et al.* (2012) issued substantial findings supporting the concept of selective covalent inhibition and specific targeting of RdRp Cys366.^6^ The study involved synthesis of inhibitors as part of a structure–activity relationship optimization program, and this then led to the discovery of 3-(1,2-dihydro-2-oxo-3-pyridinyl)-5-ethyl-1-[(2-fluoro-5-nitrophenyl)methyl]-1*H*-indole-2-carboxylic acid, otherwise referred to as compound 47 (Fig. 3). The covalent inhibitor's global experimental profile revealed good *in vitro* safety, pharmacokinetics (PK) and potency. To date, there is no experimental data from investigations of the structural dynamic entities of compound 47 and its RdRp interactions.

In this study, the selective covalent inhibition of HCV RdRp was investigated for the first time. Through extensive application of various molecular and bioinformatics tools, RdRp dynamic and structural characteristics of the free enzyme as well as a covalently-bound complex, were expressed and compared. This study emphasized the importance and necessity of selective covalent inhibition in antiviral therapy. It is hoped that this study will aid in the design and development of potent, target specific covalent inhibitors against HCV as well as a wide variety of other viruses.

## 2. Computational methodology

### 2.1 System preparation

The HCV RdRp and the three-dimensional structure of the experimental HCV inhibitor, compound 47, were obtained from the RSCB Protein Data Bank (PDB code: 3H5S and 3TYQ, respectively).^6,29^ The apo system contains only the free enzyme. The bound system's protonation states were optimized, and then directly followed by the addition of hydrogen atoms using the Protein Preparation Wizard in Maestro (Schrödinger).^30,31^ Molecular docking was utilized to envisage binding affinities and optimized conformations of compound 47 within the RdRp active site^32^ (grid box spacing of 0.375 Å and *x*, *y*, *z* dimensions of 9 × 9 × 7.72). Docking software utilized in this study included AutoDock Vina^33^ and UCSF Chimera.^34^ Compound 47 was subsequently docked in the active binding site of RdRp and the resultant complex accompanied by the most negative binding energy (kcal mol^−1^) was selected and subjected to molecular dynamics (MD) simulations.

### 2.2 Molecular dynamics simulation

The MD simulations provide a powerful tool used for the exploration of the physical activity undertaken by molecules and atoms, thus, providing a focused interpretation of the biomolecular processes of biological systems.^35^ The Amber14 MD package^36,37^ was used to perform a 200 nanoseconds (ns) MD simulation for the apo and bound system. The MD simulation was performed using the graphics processing unit (GPU) version of the Particle Mesh Ewald Molecular Dynamics (PMEMD) engine provided through Amber and the ff14SB Amber force field. The antechamber module was utilised to generate partial atomic charges for compound 47 by applying a General Amber Force Field and restrained electrostatic potential procedures.^38^ The LEaP module was utilised to neutralise and solvate both the apo and bound system systems by the addition of hydrogen atoms, and sodium and chloride ions. Because of the lack of effective protocols reported for efficient selective covalent inhibition in the literature, an in-house protocol was devised to enhance the *in silico* outcomes of the selective covalent inhibitors. A greater expansion of the protocol mentioned can be found in the papers by Khan *et al.*^39,40^ The covalent system topology and input co-ordinate files were created using Dabble.^41^ To begin with, the covalently bound system was minimized in two steps with the restraint potential of 10 Å for consideration of a solute molecule using 500 steepest descent steps, followed by 1000 steps of conjugate gradient minimization. The system was then gradually heated from 0 K to 300 K and 2.5 ns of equilibration was executed for apo and bound system stabilisation. A potential harmonic restraint of 10 kcal mol^−1^ Å^2^ for solute atoms and a Langevin thermostat with a collision frequency of 1 ps was applied to the system. The pressure and the number of atoms were kept constant to resemble an isobaric–isothermal ensemble (NPT). The pressure of both the systems were maintained at 1 bar using the Berendsen barostat^42^ and the SHAKE algorithm was utilised for the hydrogen bond constraint.^43^

### 2.3 Post analysis

The coordinates of the apo and bound systems were each saved every 1 ps and the trajectories were analysed using the CPPTRAJ and PTRAJ modules in the Amber14 package. The following analyses were performed: root mean square deviation (RMSD), root mean square fluctuations (RMSF), radius of gyration (RoG), dynamic cross correlation matrix (DCCM), template channel width, interdomain angle and solvent accessible surface analysis (SASA). Visualisation and graphical software programs were used throughout this study including visual molecular dynamics^44^ to visualize projected trajectories, Maestro^31^ software for preparation of the systems and finally Origin software (OriginLab, Northampton, MA) for the plotting and generation of graphs.

#### 2.3.1 Root mean square fluctuation (RMSF)

The fluctuation of individual enzyme residues about their average position within a given MD simulation is referred to as the RMSF.^45^ The RMSF analysis provides insights into the flexibility of various regions of the HCV RdRp upon binding of the ligand and is mathematically calculated as follows:
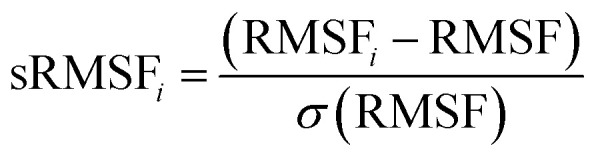
where RMSF_*i*_ represents the RMSF of the *i*^th^ residue, from which the average RMSF is subtracted. This is then divided by the RMSF's standard deviation [*σ*(RMSF)] to yield the resultant standardised RMSF (sRMSF_*i*_).

#### 2.3.2 Radius of gyration (RoG)

The RoG describes the RMSD of the atoms from the common center of gravity of a given enzyme molecule. This method of analysis allows the assessment of protein compactness along a given trajectory in a MD simulation. The next equation describes how RoG is estimated:
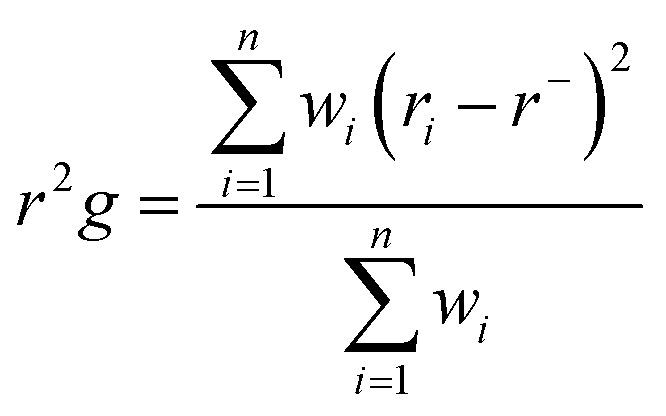
where: *r*_*i*_ is the position of the *i*^th^ atom and *r* is the center mass of atom *i*. The mean value is calculated by taking the RoG values over the number of frames in a given trajectory.^46^

#### 2.3.3 Dynamic cross correlation matrix (DCCM) analysis

Dynamic cross correlation is a method that is widely used in MD simulations to effectively quantify the correlation co-efficient of the motion between the atoms of a protein. The dynamic cross correlation (DCC) between the residue-based fluctuations during the simulation was calculated using the CPPTRAJ module incorporated in AMBER 14. The formula used to describe DCC is given as follows:
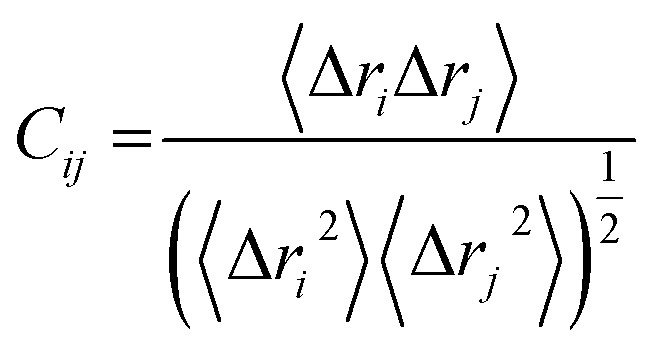
The abbreviated terms denote the following: *C*_*ij*_: cross-correlation coefficient, fully correlated (−1) to anti-correlated (+1), *i*: *i*^th^ residue, *j*: *j*^th^ residue, Δ*r*_*i*_: displacement vectors corresponding to the *i*^th^ residue, and Δ*r*_*j*_: displacement vectors corresponding to the *j*^th^ residue. The resultant DCCM of each system was constructed using Origin software.

#### 2.3.4 Thermodynamic binding free energy calculations

The MD allow admittance to free energy differences which control the underlying mechanism of all biological processes.^47^ Calculations for binding free energy (BFE) is an imperative method used to observe the in-depth binding mechanism between a protein and ligand, all-encompassing both entropic and enthalpic contributions.^48^ Estimation of the binding affinity within a docked system is calculated using the BFE with the Molecular Mechanics/Generalized Born Surface Area (MM/GBSA) technique. This method can be used to provide reproducible relative binding affinities of compounds with good accuracy and requires substantially less computational resources in comparison to a full-scale MD free energy perturbation/thermodynamic simulation.^49^ In this study, BFE was averaged over 20 000 snapshots, which were generated from the 200 ns trajectory. The explicit solvent used in the MD simulation was discarded and substituted with a dielectric continuum as per the MM/GBSA protocol.^50^ Changes in each term between the apo and bound states were calculated and contributed to the total relative BFE.^49,50^ The MM force fields were then used to calculate energy contributions from the atomic coordinates of the ligand, receptor and complex in a gaseous phase. The equations shown next, present the process in which the binding free energies (Δ*G*) were assessed:

1 Δ*G*_bind_ = Δ*G*_complex_ − Δ*G*_receptor_ − Δ*G*_ligand_

2 Δ*G*_bind_ = *E*_gas_ + *G*_sol_ − *T*Δ*S*

3 *E*_gas_ = *E*_int_ + *E*_vdw_ + *E*_ele_

4 *G*_sol_ + *G*_GB_ + *G*_SA_

5 *G*_SA_ + γSASA

where Δ*G*_bind_ is gas-phase summation, *E*_gas_ is the gas-phase energy and the solvation energy, *G*_sol_, is less the entropy (*T*Δ*S*) term. The *E*_gas_ is the sum of internal energy, *E*_int_, van der Waals (vdW) energy, *E*_vdW_ and electrostatic energy, *E*_ele_. The total solvation energy is calculated using a summation of the total energy contributions of polar and non-polar states (*G*_GB_ and *G*_SA_, respectively). The *G*_SA_ is calculated using the solvent accessible surface area, generated by a water probe radius of 1.4 Å. Resolution of the *G*_GB_ equation allows the determination of the energy contributions of the polar states. The total entropy of the solute is denoted as ‘*S*’ and the temperature as ‘*T*’. The solute and solvent dielectric constants are set to 1 and 80, respectively.^51,52^

The MM/GBSA method was also used to calculate the final energy per residue decomposition.^53^ Concerning the estimated relative BFEs, the degree of accuracy may be enhanced if the terms in the equation, particularly those in eqn (2), are averaged over several MD snapshots but typically this depends on the area of research.^50^ Conduction of separate MD simulations for the receptor, ligand and complex will produce more accurate BFE results, however, it requires greater computational resources which were not readily available for this study.^49,50^ Although the MM/GBSA technique lacks the required accuracy for absolute BFE estimates,^51,54^ several previous reports including studies from this research group have successfully used the MM/GBSA approach in obtaining relative BFE for MD simulated systems which have been validated with experimental findings.^38,49,55–59^ To obtain individual residue contributions to the total BFE profile of compound 47 with the HCV RdRp, per residue free energy decomposition was performed at an atomic level for all the important residues using the MM/GBSA method of Amber14.

## 3. Results and discussion

### 3.1 Stability of NS5B RdRp apo and bound system

**Fig. 4:**
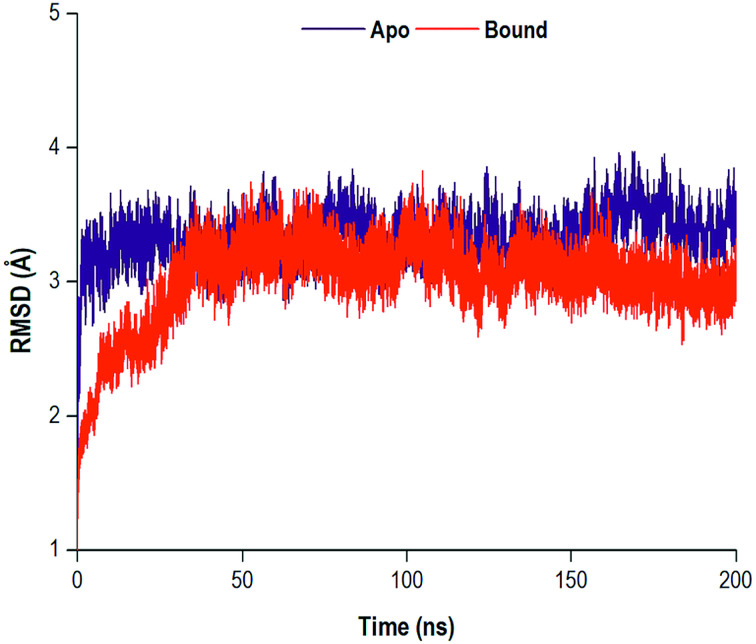
RMSD plot of C-α atoms of the apo and bound systems.

The MD simulations yielded comprehensive insights into the structure and dynamics of a biological system. The convergence of a system may be reached as the system gains stability, often occurring in an increasingly longer MD simulation. Therefore, the structural dynamic examination of a system is highly dependent on the timescale of the MD simulation.^60^ Throughout the 200 ns simulation, the RMSD of the C-α (alpha carbon) backbone were observed for both the apo and bound systems. The RMSD analysis was used to graphically monitor system convergence.^61^ Both the apo and bound systems converged at approximately 30 ns and 35 ns, respectively (Fig. 4).

### 3.2 Conformational fluctuations of NS5B RdRp

**Fig. 5:**
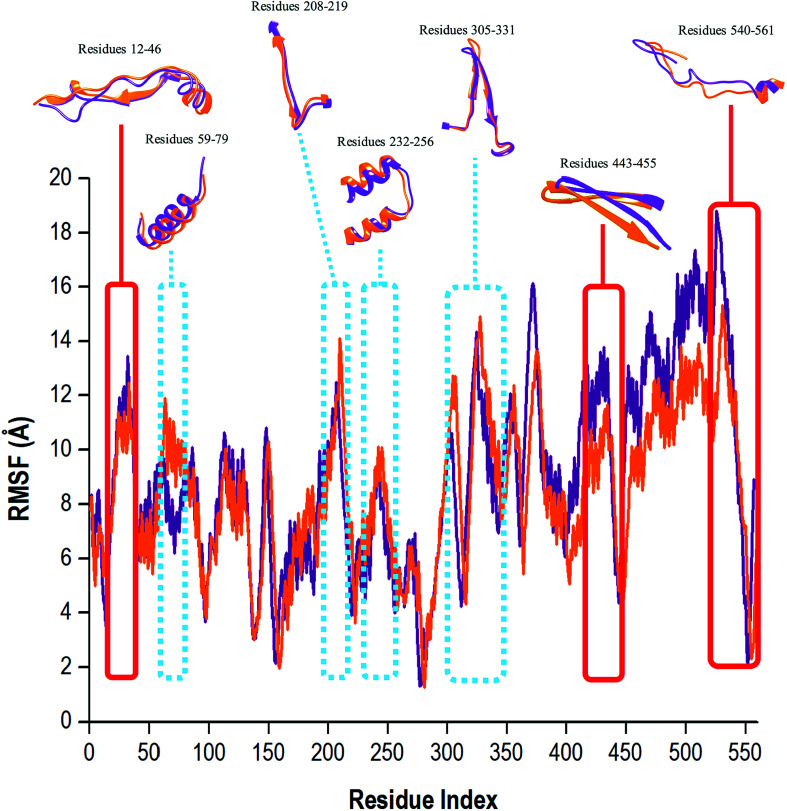
Residue-based average C-α fluctuations of the apo and bound conformation of HCV RdRp throughout the 200 ns MD simulation.

To determine whether the binding of compound 47 affected dynamic residue behavior, the RMSF values of the apo and bound systems were determined. The RMSF with respect to the averaged MD simulation conformation was used as a means to demonstrate the differences arising in the flexibility of the residues. The RMSF of all residues were calculated for structure backbone flexibility and are shown in Fig. 5.

Higher C-α fluctuation values signified more flexible movements in relation to the average position of the residues, and in contrast, reduced values express restricted fluctuations during the simulation.^62^ In Fig. 5, the apo RdRp presented with an overall higher residual fluctuation (8.97 Å) when compared to the bound system (8.47 Å). This degree of high flexibility was in agreement with results in other papers in the literature, whereby this property enabled the enzyme to accept single-stranded RNA templates, and in doing so, the enzyme can effectively propagate viral replication. This finding strongly indicated that the binding of compound 47 to the RdRp, lowered dynamic residual fluctuations of the enzyme, thus inducing stability of the bound state.

To effectively understand a protein's function at an atomic level, experimental analysis of the structure was required. It is important to note that the functional properties of a protein were determined not only by its relative structural rigidity but also by its dynamic behavior.^63,64^ The binding site residues of compound 47 within 5 Å were: Arg158, Phe193, Pro197, Arg200, Asn316, Asp318, Asp319, Thr364, Ser365, Ser367, Ser368, Leu384, Arg386, Arg399, Ser407, Gly410, Asn411, Met414, Tyr415, Gln446, Ile447, Tyr448, Gly449, Ala450, Tyr555 and Ser556. Compound 47 was bound covalently to Cys366 located in the palm domain of the RdRp within the active binding site. In Fig. 5, close observation revealed that the unbound RdRp experienced a great degree of flexibility as the regions assumed differing conformations in accordance with the opening and closing of the active binding site. Upon binding of compound 47, the residual fluctuations of RdRp were lowered and regions greatly affected by the covalent binding encompassed residues 12–46, 443–455 and 530–561. During the 200 ns MD simulation, the covalently bound system did experience occasions of elevated C-α fluctuations. The regions with the most notable fluctuations were residues 59–79, 208–219, 232–256 and 305–331 (Fig. 5, highlighted in blue). This is because the RdRp underwent conformational adjustments to accommodate compound 47 binding and subsequent ligand–enzyme interactions. In essence, compound 47 caused a certain degree of interference with the enzyme's conformation, which was not good for optimum enzyme functionalities, leading to interruptions in or possibly extermination of downstream activities important in viral replication.^8,16^

**Fig. 6:**
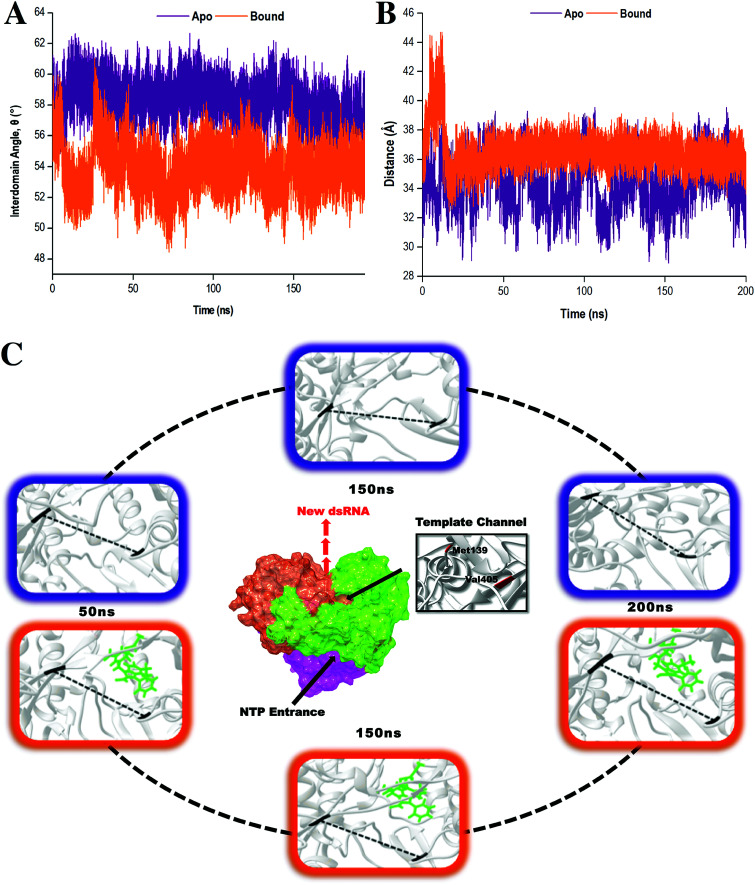
Distance analysis demonstrating the conformational changes of the TCW and interdomain angle upon compound 47 binding. (A) The interdomain angle was computed by measuring the angle between the centre of the masses of the finger, thumb and palm domains. (B) The width was calculated by measuring the distance between the C-α atoms of residues Met139 and Val405. (C) Structural dynamic movements of the TCW in the apo (purple) and bound (orange, ligand highlighted in green) states of HCV RdRp throughout a 200 ns MD simulation. The dashed line in the highlighted images represents the distance between the C-α atoms of the two residues.

To provide further insight into the impact of compound 47 binding during conformational sampling, template channel width (TCW) and interdomain angles were investigated. Narrower widths and smaller angles corresponded to a more closed enzyme conformation.^65^ In Fig. 6A and B, it was observed that the apo system displayed an average interdomain angle of 58.92° and experienced fluctuations in TCW ranging between 28.15 Å and 39.55 Å. In contrast, the covalently bound system exhibited a more compact structure with an average interdomain angle of 53.92° and a narrower TCW range of 33.39–39.19 Å. Overall, compound 47 binding limits the conformational sampling of HCV RdRp, shifting the enzyme to a more stabilized closed conformation. Compound 47 allowed the thumb and finger subdomains to come into proximity to one another thereby decreasing the TCW, subsequently altering the interdomain angle. In doing so, this created a difficult task for the RNA template to gain access to the template channel and the active binding site.^66^ Based on these findings, it is believed that compound 47 prevents the transitions between closed and open conformations, which are imperative for RdRp functioning in replication, leading to inadequate and inefficient execution of RNA elongation and subsequent viral replication.^7,67^

**Fig. 7:**
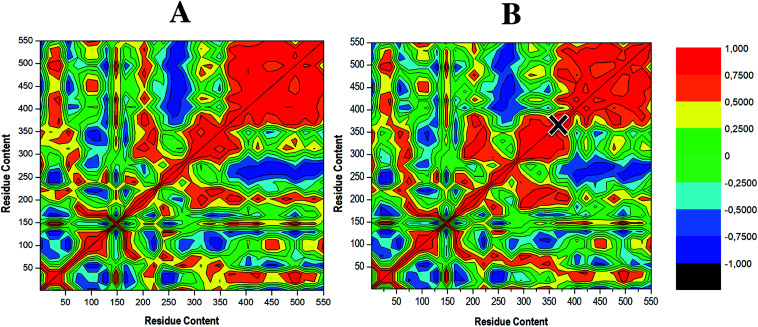
Dynamic cross-correlation matrix presenting correlation of residues in the apo (A) and compound 47-bound system (B). The status of correlated motions is deduced by the colour scale on the right, the black cross indicates Cys366 to which compound 47 is covalently bound.

To determine the occurrence of correlated dynamics, DCCM analysis was performed. This method computes the position of the C-α atoms throughout each independent 200 ns MD simulation.^68^ The colours red to yellow represent highly positive correlated C-α movements. In contrast, negative or anti-correlated movements are represented as colours blue to black. Whereas similar correlated motions occur in both cases, the apo enzyme shows higher levels of correlation (red region), further justified by observing a higher occupancy of positive cross-correlation coefficient, as shown in Fig. 7A. This correlation decreases upon ligand binding (Fig. 7B), suggesting selective covalent inhibitor, compound 47, induced a reduction in fluctuations of RdRp C-α atoms. Residues 246–282 and 380–550 which correspond to the finger and thumb domains, respectively, displayed anti-correlated movement (dark blue region) in the apo and to a slightly lesser degree in the bound system, supporting the residue fluctuations in Fig. 5. These domain motions serve a fundamental importance as they facilitate an RdRp closed “active” conformation, which allows for *de novo* synthesis of RNA. Therefore, reduction when compound 47 is bound would be consistent with the inability of the enzyme to efficiently execute viral replication as the RdRp will not be adequately equipped to accept and bind the RNA template, thus hindering the process of viral replication.^7^

### 3.3 Solvent accessible surface area and radius of gyration

**Fig. 8:**
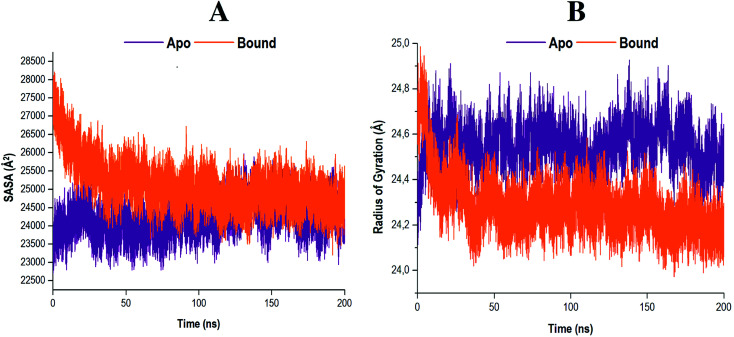
(A) Solvent accessible surface area of apo and compound 47 bound RdRp. (B) Radiuses of gyration of both systems were measured over a 200 ns simulation. Data obtained were represented as a plot displaying the differences arising in radius deviation between the apo and covalent systems.

To further clarify the impact of compound 47 binding on the structure of RdRp, the SASA was evaluated. Solvent accessibility quantifies the area of a protein obtainable by or exposed to solvent molecules.^69,70^ The SASA analyses of both systems were conducted and the results obtained are presented in Fig. 8A.

The results indicated that the apo system maintained a consistent residual exposure to solvents throughout the 200 ns MD simulation. In contrast, the bound system exhibited a progressive decline in the area accessible to solvents upon ligand binding. This may serve as an indication that following compound 47 binding to the RdRp, the structural integrity of the protein was compromised. The active site residues underwent restructuring, and in the process diminished the area, which was exposed to solvents. Consequently, these actions may result in the functional loss of proper enzyme activity.^71^

The RoG measures the compactness of a protein whilst concurrently providing insight into the biological system's stability.^46,72,73^ The comparative RoG for the apo and bound systems is shown in Fig. 8B. From the relevant figure, it is observed that the RoG value for both the apo and bound systems are fairly similar, with an average value of 24.55 Å and 24.28 Å, respectively. These findings suggest that compound 47 binding to RdRp Cys366 induced a conformational shift to a more compact structure, despite there only being a 0.27 Å change, ultimately forming a relatively stable enzyme.

### 3.4 Ligand interaction with HCV NS5B RdRp

**Fig. 9:**
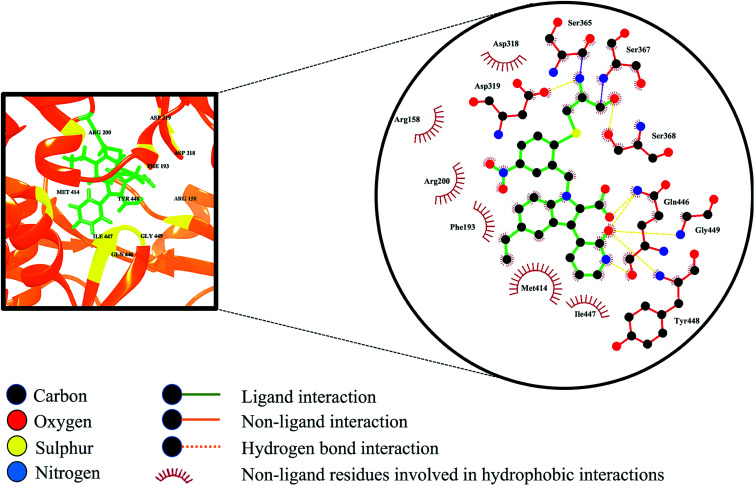
Ligand–residue interaction diagram of compound 47, inside the active site of the palm domain of the RdRp. Orange dashed lines denote hydrogen bond interactions.

Selective covalent inhibition disrupts the atomic backbone dynamics of the RdRp through movement of the regulatory elements such as the thumb domain, β-loop and the C-terminal tail. Normal physiological structure-based dynamics of the enzyme requires these important modulatory components for efficient viral transcription and replication. As observed in Fig. 9, the indole core (C_8_H_7_N) of the inhibitor as well as the C-3 pyridone ring are in close contact with Met414. Generally, the active site adopts a favourable morphology to allow influential elements such as the β-loop, a component vital for *de novo* initiation, to swing away from the active site thereby allowing the elongation of the RNA product. When compound 47 undergoes binding, the C-3 pyridone ring of the inhibitor forms three hydrogen interactions with the backbone of the β-loop residues Gln446, Tyr448 and Gly449. These bond interactions induce the descent of the β-loop further into the palm site thereby disrupting the structural conformation of RdRp, giving rise to loss of structural integrity. Ultimately, these consequences may result in the overall prevention of RdRp enzymatic activity.^74^

### 3.5 Free binding energy calculations

The MM/GBSA method is extensively used to approximate the BFE of an inhibitor at an atomic level.^75^ The MM/GBSA method was used to evaluate the total binding energy contributions of compound 47 to the RdRp as shown in Table 1.

**Table 1:** Summary of MM/GBSA-based binding free energy contributions to the compound 47-HCV RdRp complex^a^

	Energy components (kcal mol^−1^)				
	Δ*E*_vdW_	Δ*E*_elec_	Δ*G*_gas_	Δ*G*_solv_	Δ*G*_bind_
HCV RdRp	−4537.81 ± 26.86	−35 742.39 ± 139.77	−4144.35 ± 146.43	−7040.47 ± 115.57	−11 184.82 ± 79.80
Compound 47	−7.30 ± 2.0	−9.39 ± 2.45	78.08 ± 6.48	−37.53 ± 1.20	40.55 ± 6.36
Complex	−47.77 ± 2.92	−160.62 ± 7.68	−129.92 ± 8.14	60.94 ± 5.92	−68.98 ± 4.55

a Δ*E*_elec_: electrostatic, Δ*E*_vdW_: van der Waals, Δ*G*_bind_: calculated total free binding energy, Δ*G*_gas_: gas phase interaction, and Δ*G*_solv_: solvation free energy.

The calculated free binding energies provide conclusive evidence at the molecular level. This technique delivers good data which can be used to lay the groundwork that can improve the design and discovery of small inhibitory molecules that possess enhanced ligand binding properties. The calculations are dynamic, inexpensive and can be conducted by members of the scientific community as the process is programmed using an external interface server and the required software is easily accessible online. The calculated free binding energy of compound 47 and the RdRp system was −68.98 kcal mol^−1^. Interaction forces such as vdW (−47.77 ± 2.92 kcal mol^−1^) and electrostatic (−160.62 ± 7.68 kcal mol^−1^) contribute greatly towards the total binding energy of compound 47 to RdRp. The hydrophobic residues lining the binding site pocket contribute significantly to the covalently bound system's free binding energy.

### 3.6 Per-residue interaction energy decomposition analysis

**Fig. 10:**
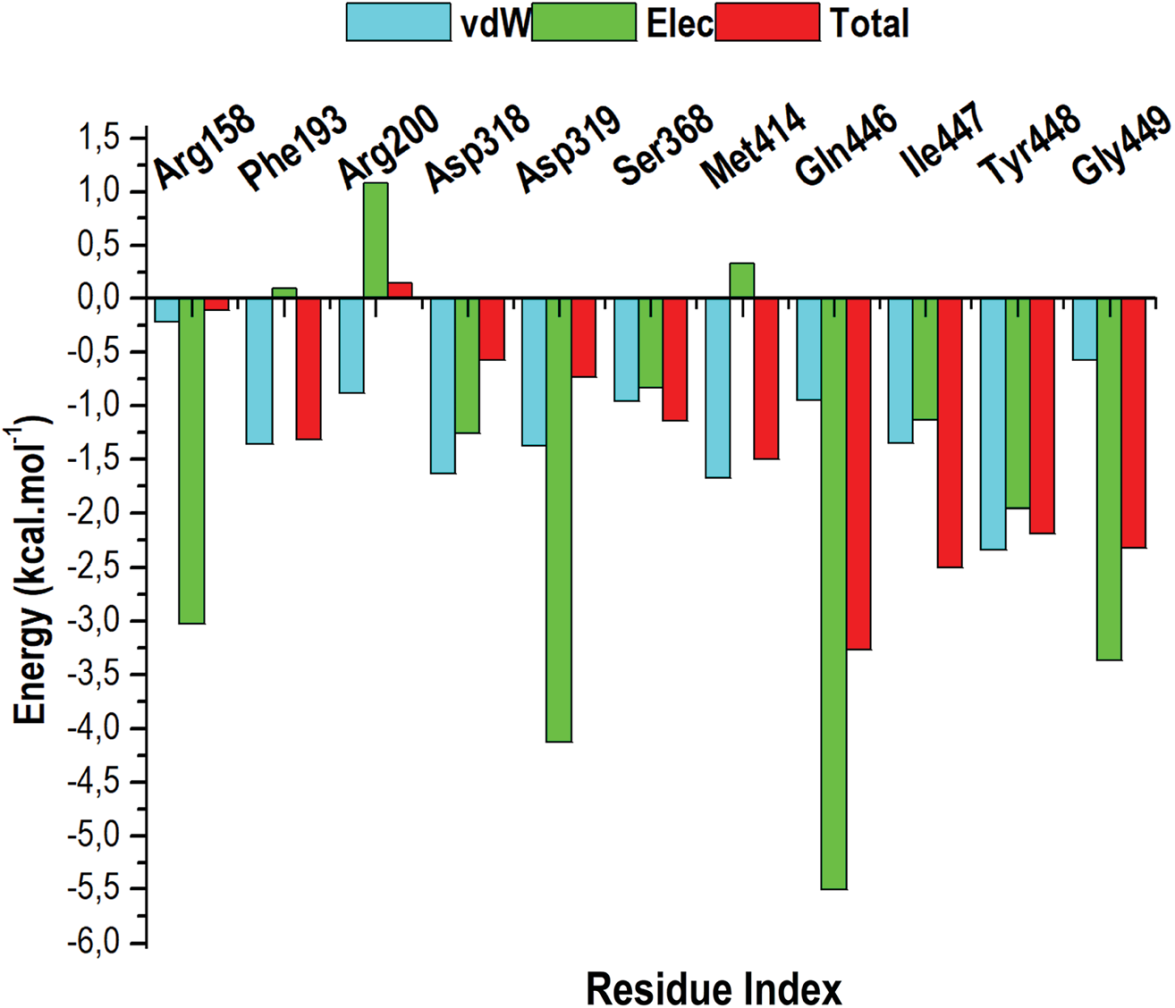
The per-residue energy decomposition analysis of compound 47 bound HCV RdRp.

The total BFE for compound 47 was decomposed into individual residue-based contributions using the MM/GBSA approach. The vdW and electrostatic (elec) interaction contributions relative to the BFE of compound 47 to RdRp was estimated to acquire an insight into which residue and energy constituents had an overall greater impact on the total binding energy (total). Per-residue energy decomposition analysis was executed and the results are presented as Fig. 10.

Intermolecular interactions between the residues of the active site facilitate the binding and stabilisation of compound 47 in the hydrophobic pocket of RdRp. As observed in Fig. 10, residues that contributes the most energy towards the complex include Arg158 [−3.02 kcal mol^−1^ (elec)], Asp319 [−4.125 kcal mol^−1^ (elec)], Gln446 [−5.51 kcal mol^−1^ (elec)], Tyr448 [−2.35 kcal mol^−1^ (vdW)], [−1.96 kcal mol^−1^ (elec)] and Gly449 [−3.37 kcal mol^−1^ (elec)]. The binding site residues that contributed less energy towards the complex were: Phe193 [−1.35 kcal mol^−1^ (vdw), 0.10 kcal mol^−1^ (elec)], Arg200 [−1.08 kcal mol^−1^ (vdW), 1.08 kcal mol^−1^ (elec)], Asp318 [−1.64 kcal mol^−1^ (vdW), −1.23 kcal mol^−1^ (elec)], Ser368 [−0.96 kcal mol^−1^ (vdW), −0.83 kcal mol^−1^ (elec)], and Met414 [−1.68 kcal mol^−1^ (vdW), −0.33 kcal mol^−1^ (elec)]. From Fig. 10, it can be deduced that electrostatic interactions from residues 158, 319, 446, 449 and vdW interactions from residues 448 and 414 contributed to the high energy interaction of the covalent system (Δ*G*_bind_: −68.98 kcal mol^−1^).

## 4. Conclusion

The HCV RdRp has a pivotal role as the driving force behind viral transcription and replication. Currently available drugs exhibit rigid pharmacodynamic profiles that require optimization for efficient viral inhibition in a safe yet effective manner. The conceptualization of selective covalent inhibition brought a new, targeted approach to HCV antiviral therapeutics. Compound 47, an indole core small inhibitor, binds covalently to Cys366 of the RdRp. The consequential shift in conformation prompted by the selective covalent binding was led by modifications in enzyme flexibility, correlated dynamics and intermolecular bonding. Taken together, dynamic and structural effects of compound 47 binding disseminate throughout the RdRp, affecting the structural integrity of the enzyme. This may lead to the loss of appropriate RdRp functioning, ultimately preventing viral replication and propagation. Therefore, it can be concluded that selective covalent inhibition is a promising approach that has the potential to facilitate future potent anti-HCV therapeutic strategies.

## Conflicts of interest

The authors declare no potential conflicts of interest.

## Supplementary Material

## References

[cit1] Kazakov T., Yang F., Ramanathan H. N., Kohlway A., Diamond M. S., Lindenbach B. D. (2015). PLoS Pathog..

[cit2] Choo Q. L., Kuo G., Weiner A. J., Overby L. R., Bradley D. W., Houghton M. (1989). Science (80- ).

[cit3] Karoney M. J., Siika A. M., Karoney M. J. (2013). Pan African Med. J..

[cit4] Barakat K. H., Law J., Prunotto A., Magee W. C., Evans D. H., Tyrrell D. L., Tuszynski J., Houghton M. (2013). J. Chem. Inf. Model..

[cit5] Sun S., Rao V. B., Rossmann M. G. (2010). Curr. Opin. Struct. Biol..

[cit6] Chen K. X., Lesburg C. A., Vibulbhan B., Yang W., Chan T. Y., Venkatraman S., Velazquez F., Zeng Q., Bennett F., Anilkumar G. N., Duca J., Jiang Y., Pinto P., Wang L., Huang Y., Selyutin O., Gavalas S., Pu H., Agrawal S., Feld B., Huang H. C., Li C., Cheng K. C., Shih N. Y., Kozlowski J. A., Rosenblum S. B., Njoroge F. G. (2012). J. Med. Chem..

[cit7] Eltahla A. A., Tay E., Douglas M. W., White P. A. (2014). Antimicrob. Agents Chemother..

[cit8] Sesmero E., Thorpe I. F. (2015). Viruses.

[cit9] Wei Y., Li J., Qing J., Huang M., Wu M., Gao F., Li D., Hong Z., Kong L., Huang W., Lin J. (2016). PLoS One.

[cit10] Barreca M. L., Iraci N., Manfroni G., Cecchetti V. (2011). Future Med. Chem..

[cit11] Chen K. X., Vibulbhan B., Yang W., Sannigrahi M., Velazquez F., Chan T. Y., Venkatraman S., Anilkumar G. N., Zeng Q., Bennet F., Jiang Y., Lesburg C. A., Duca J., Pinto P., Gavalas S., Huang Y., Wu W., Selyutin O., Agrawal S., Feld B., Huang H. C., Li C., Cheng K. C., Shih N. Y., Kozlowski J. A., Rosenblum S. B., Njoroge F. G. (2012). J. Med. Chem..

[cit12] Dousson C. B. (2018). Antiviral Chem. Chemother..

[cit13] Lawitz E., Buti M., Vierling J. M., Almasio P. L., Bruno S., Ruane P. J., Hassanein T. I., Muellhaupt B., Pearlman B., Jancoriene L., Gao W., Huang H. C., Shepherd A., Tannenbaum B., Fernsler D., Li J. J., Grandhi A., Liu H., Su F. H., Wan S., Dutko F. J., Nguyen B. Y. T., Wahl J., Robertson M. N., Barr E., Yeh W. W., Plank R. M., Butterton J. R., Yoshida E. M. (2017). Lancet Gastroenterol. Hepatol..

[cit14] Wyles D., Wedemeyer H., Ben-Ari Z., Gane E. J., Hansen J. B., Jacobson I. M., Laursen A. L., Luetkemeyer A., Nahass R., Pianko S., Zeuzem S., Jumes P., Huang H. C., Butterton J., Robertson M., Wahl J., Barr E., Joeng H. K., Martin E., Serfaty L. (2017). Hepatology.

[cit15] Devogelaere B., Berke J. M., Vijgen L., Dehertogh P., Fransen E., Cleiren E., Van Der Helm L., Nyanguile O., Tahri A., Amssoms K., Lenz O., Cummings M. D., Clayton R. F., Vendeville S., Raboisson P., Simmen K. A., Fanning G. C., Lin T. I. (2012). Antimicrob. Agents Chemother..

[cit16] Eltahla A. A., Luciani F., White P. A., Lloyd A. R., Bull R. A. (2015). Viruses.

[cit17] Li J., Liu X., Li S., Wang Y., Zhou N., Luo C., Luo X., Zheng M., Jiang H., Chen K. (2013). Int. J. Mol. Sci..

[cit18] Buxton I., Benet L. (2011). Goodman Gilman’s Pharmacol. Basis Ther..

[cit19] Kumthip K., Maneekarn N. (2015). Virol. J..

[cit20] Echeverria N., Moratorio G., Cristina J., Moreno P. (2015). World J. Hepatol..

[cit21] Fonseca-Coronado S., Escobar-Gutiérrez A., Ruiz-Tovar K., Cruz-Rivera M. Y., Rivera-Osorio P., Vazquez-Pichardo M., Carpio-Pedroza J. C., Ruíz-Pacheco J. A., Cazares F., Vaughan G. (2012). J. Clin. Microbiol..

[cit22] Johnson D. S., Weerapana E., Cravatt B. F. (2010). Future Med. Chem..

[cit23] Bauer R. A. (2015). Drug Discov. Today.

[cit24] Lee G., Piper D. E., Wang Z., Anzola J., Powers J., Walker N., Li Y. (2006). J. Mol. Biol..

[cit25] Hagel M., Niu D., St Martin T., Sheets M. P., Qiao L., Bernard H., Karp R. M., Zhu Z., Labenski M. T., Chaturvedi P., Nacht M., Westlin W. F., Petter R. C., Singh J. (2011). Nat. Chem. Biol..

[cit26] Hallenbeck K. K., Turner D. M., Renslo A. R., Arkin M. R. (2017). Curr. Top. Med. Chem..

[cit27] McWilliam H., Li W., Uludag M., Squizzato S., Park Y. M., Buso N., Cowley A. P., Lopez R. (2013). Nucleic Acids Res..

[cit28] Bateman A., Martin M. J., O'Donovan C., Magrane M., Alpi E., Antunes R., Bely B., Bingley M., Bonilla C., Britto R., Bursteinas B., Bye-AJee H., Cowley A., Da Silva A., De Giorgi M., Dogan T., Fazzini F., Castro L. G., Figueira L., Garmiri P., Georghiou G., Gonzalez D., Hatton-Ellis E., Li W., Liu W., Lopez R., Luo J., Lussi Y., MacDougall A., Nightingale A., Palka B., Pichler K., Poggioli D., Pundir S., Pureza L., Qi G., Rosanoff S., Saidi R., Sawford T., Shypitsyna A., Speretta E., Turner E., Tyagi N., Volynkin V., Wardell T., Warner K., Watkins X., Zaru R., Zellner H., Xenarios I., Bougueleret L., Bridge A., Poux S., Redaschi N., Aimo L., ArgoudPuy G., Auchincloss A., Axelsen K., Bansal P., Baratin D., Blatter M. C., Boeckmann B., Bolleman J., Boutet E., Breuza L., Casal-Casas C., De Castro E., Coudert E., Cuche B., Doche M., Dornevil D., Duvaud S., Estreicher A., Famiglietti L., Feuermann M., Gasteiger E., Gehant S., Gerritsen V., Gos A., Gruaz-Gumowski N., Hinz U., Hulo C., Jungo F., Keller G., Lara V., Lemercier P., Lieberherr D., Lombardot T., Martin X., Masson P., Morgat A., Neto T., Nouspikel N., Paesano S., Pedruzzi I., Pilbout S., Pozzato M., Pruess M., Rivoire C., Roechert B., Schneider M., Sigrist C., Sonesson K., Staehli S., Stutz A., Sundaram S., Tognolli M., Verbregue L., Veuthey A. L., Wu C. H., Arighi C. N., Arminski L., Chen C., Chen Y., Garavelli J. S., Huang H., Laiho K., McGarvey P., Natale D. A., Ross K., Vinayaka C. R., Wang Q., Wang Y., Yeh L. S., Zhang J. (2017). Nucleic Acids Res..

[cit29] de Vicente J., Hendricks R. T., Smith D. B., Fell J. B., Fischer J., Spencer S. R., Stengel P. J., Mohr P., Robinson J. E., Blake J. F., Hilgenkamp R. K., Yee C., Adjabeng G., Elworthy T. R., Li J., Wang B., Bamberg J. T., Harris S. F., Wong A., Leveque V. J. P., Najera I., Le Pogam S., Rajyaguru S., Ao-Ieong G., Alexandrova L., Larrabee S., Brandl M., Briggs A., Sukhtankar S., Farrell R. (2009). Bioorganic Med. Chem. Lett..

[cit30] Bhachoo J. and Beuming T., in Methods in Molecular Biology, 2017, vol. 1561, pp. 235–254 10.1007/978-1-4939-6798-8_14 28236242

[cit31] Madhavi Sastry G., Adzhigirey M., Day T., Annabhimoju R., Sherman W. (2013). J. Comput. Aided. Mol. Des..

[cit32] Yang Z., Lasker K., Schneidman-Duhovny D., Webb B., Huang C. C., Pettersen E. F., Goddard T. D., Meng E. C., Sali A., Ferrin T. E. (2012). J. Struct. Biol..

[cit33] Trott O., Olson A. J. (2010). J. Comput. Chem..

[cit34] Pettersen E. F., Goddard T. D., Huang C. C., Couch G. S., Greenblatt D. M., Meng E. C., Ferrin T. E. (2004). J. Comput. Chem..

[cit35] Fogolari F., Corazza A., Toppo S., Tosatto S. C. E., Viglino P., Ursini F., Esposito G. (2012). J. Biomed. Biotechnol..

[cit36] Case D. a., Cheatham I. T. E., Darden T., Gohlke H., Luo R., Merz J. K. M., Onufriev a., Simmerling C., Wang B., Woods R. (2005). J. Comput. Chem..

[cit37] Salomon-Ferrer R., Case D. A., Walker R. C. (2013). Wiley Interdiscip. Rev.: Comput. Mol. Sci..

[cit38] Ramharack P., Oguntade S., Soliman M. E. S. (2017). RSC Adv..

[cit39] Khan S., Bjij I., Betz R. M., Soliman M. E. (2018). Future Med. Chem..

[cit40] Khan S., Bjij I., Oluto F., Soliman M. E. S. (2018). Future Med. Chem..

[cit41] Betz R. , Dabble, 2017, https://dabble.robinbetz.com/

[cit42] Mhlongo N. N., Ebrahim M., Skelton A. A., Kruger H. G., Williams I. H., Soliman M. E. S. (2015). RSC Adv..

[cit43] Gonnet P. (2007). J. Comput. Phys..

[cit44] Humphrey W., Dalke A., Schulten K. (1996). J. Mol. Graphics.

[cit45] Bornot A., Etchebest C., De Brevern A. G. (2011). Proteins: Struct., Funct., Bioinf..

[cit46] Lobanov M. I., Bogatyreva N. S., V Galzitskaia O. (2008). Mol. Biol..

[cit47] Gapsys V., Michielssens S., Peters J. H., de Groot B. L., Leonov H. (2015). Methods Mol. Biol..

[cit48] Schauperl M., Czodrowski P., Fuchs J. E., Huber R. G., Waldner B. J., Podewitz M., Kramer C., Liedl K. R. (2017). J. Chem. Inf. Model..

[cit49] Agoni C., Ramharack P., Soliman M. E. S. (2018). Future Med. Chem..

[cit50] Hayes J. M. and Archontis G., in Molecular Dynamics – Studies of Synthetic and Biological Macromolecules, 2011

[cit51] Hou T., Wang J., Li Y., Wang W. (2011). J. Chem. Inf. Model..

[cit52] Zhang X., Perez-sanchez H., Lightstone F. C. (2017). Curr. Top. Med. Chem..

[cit53] Miller B. R., McGee T. D., Swails J. M., Homeyer N., Gohlke H., Roitberg A. E. (2012). J. Chem. Theory Comput..

[cit54] Singh N., Warshel A. (2010). Proteins: Struct., Funct., Bioinf..

[cit55] Ramharack P., Soliman M. E. S. (2018). J. Biomol. Struct. Dyn..

[cit56] Machaba K. E., Mhlongo N. N., Dokurugu Y. M., Soliman M. E. (2017). Future Med. Chem..

[cit57] Ndagi U., Mhlongo N. N., Soliman M. E. (2017). Mol. BioSyst..

[cit58] Oguntade S., Ramharack P., Soliman M. E. (2017). Future Virol..

[cit59] Appiah-Kubi P., Soliman M. E. S. (2016). J. Biomol. Struct. Dyn..

[cit60] Galindo-Murillo R., Roe D. R., Cheatham T. E. (2015). Biochim. Biophys. Acta, Gen. Subj..

[cit61] Hess B. (2002). Phys. Rev. E: Stat. Phys., Plasmas, Fluids, Relat. Interdiscip. Top..

[cit62] Kumar C. V., Swetha R. G., Anbarasu A., Ramaiah S. (2014). Adv. Bioinf..

[cit63] Henzler-Wildman K., Kern D. (2007). Nature.

[cit64] Yang L. Q., Sang P., Tao Y., Fu Y. X., Zhang K. Q., Xie Y. H., Liu S. Q. (2014). J. Biomol. Struct. Dyn..

[cit65] Davis B. C., Brown J. A., Thorpe I. F. (2015). Biophys. J..

[cit66] Davis B. C., Brown J. A., Thorpe I. F. (2015). Biophys. J..

[cit67] Brown J. A., Thorpe I. F. (2015). Biochemistry.

[cit68] Ndagi U., Mhlongo N. N., Soliman M. E. (2017). Mol. BioSyst..

[cit69] Richmond T. J. (1984). J. Mol. Biol..

[cit70] McGillewie L., Soliman M. E. (2016). Mol. BioSyst..

[cit71] Boyce S. E., Tirunagari N., Niedziela-Majka A., Perry J., Wong M., Kan E., Lagpacan L., Barauskas O., Hung M., Fenaux M., Appleby T., Watkins W. J., Schmitz U., Sakowicz R. (2014). PLoS One.

[cit72] Sindhu T., Srinivasan P. (2015). RSC Adv..

[cit73] McGillewie L., Soliman M. E. (2016). Mol. BioSyst..

[cit74] Li J., Johnson K. A. (2016). J. Biol. Chem..

[cit75] Genheden S., Ryde U. (2015). Expert Opin. Drug Discovery.

